# Crystal structures of the isotypic complexes bis­(morpholine)­gold(I) chloride and bis­(morpholine)­gold(I) bromide^1^

**DOI:** 10.1107/S2056989023009702

**Published:** 2023-11-16

**Authors:** Cindy Döring, Peter G. Jones

**Affiliations:** a Institut für Anorganische und Analytische Chemie, Technische Universität Braunschweig, Hagenring 30, D-38106 Braunschweig, Germany; Universität Greifswald, Germany

**Keywords:** crystal structure, gold, morpholine, secondary inter­actions

## Abstract

The compounds bis­(morpholine-κ*N*)gold(I) chloride, [Au(C_4_H_9_NO)_2_]Cl, **1**, and bis­(morpholine-κ*N*)gold(I) bromide, [Au(C_4_H_9_NO)_2_]Br, **2**, crystallize isotypically in space group *C*2/*c* with *Z* = 4. The gold atoms, which are axially positioned at the morpholine rings, lie on inversion centres (so that the N—Au—N coordination is exactly linear) and the halide anions on twofold axes. The residues are connected by a classical hydrogen bond N—H⋯halide and by a short gold⋯halide contact to form a layer structure parallel to the *bc* plane. The morpholine oxygen atom is not involved in classical hydrogen bonding.

## Abstract 2

In the title compounds, the gold atoms lie on inversion centres and are linearly coordinated by two morpholine ligands. The halide anions lie on twofold axes. Hydrogen bonds and Au⋯halide contacts lead to a layer structure.

## 1. Chemical context

We are inter­ested in the synthesis and, particularly, the structures of amine complexes of gold halides and pseudohalides. These structures often display packing features such as aurophilic inter­actions (reviewed by Schmidbaur & Schier, 2008 ▸, 2012 ▸), hydrogen bonding (sometimes involving metal-bonded halogens; Brammer, 2003 ▸), gold⋯halogen contacts or halogen⋯halogen contacts (see *e.g*. Metrangelo, 2008 ▸). Background material, including an extensive summary of our previous investigations, can be found in the previous article of this series (Döring & Jones, 2023 ▸), which presented complexes involving piperidine and pyrrolidine complexes. The ligand morpholine, C_4_H_9_NO, (sometimes referred to as 1,4-oxazin­ane or tetra­hydro-1,4-oxazine, although morpholine is the preferred IUPAC name; here abbreviated in formulae as ‘morph’) is closely similar to piperidine (both are six-membered rings involving secondary amine functions), but the presence of the oxygen atom in the ring might lead to additional possibilities for hydrogen bonding. Here we present the structures of the isotypic complexes bis­(morpholine)­gold(I) chloride, [Au(morph)_2_]Cl, **1** and bis­(morpholine)­gold(I) bromide, [Au(morph)_2_]Br, **2**. We have already reported the synthesis of **1** (Ahrens *et al.*, 1999 ▸), but the structure was not determined at that time. 
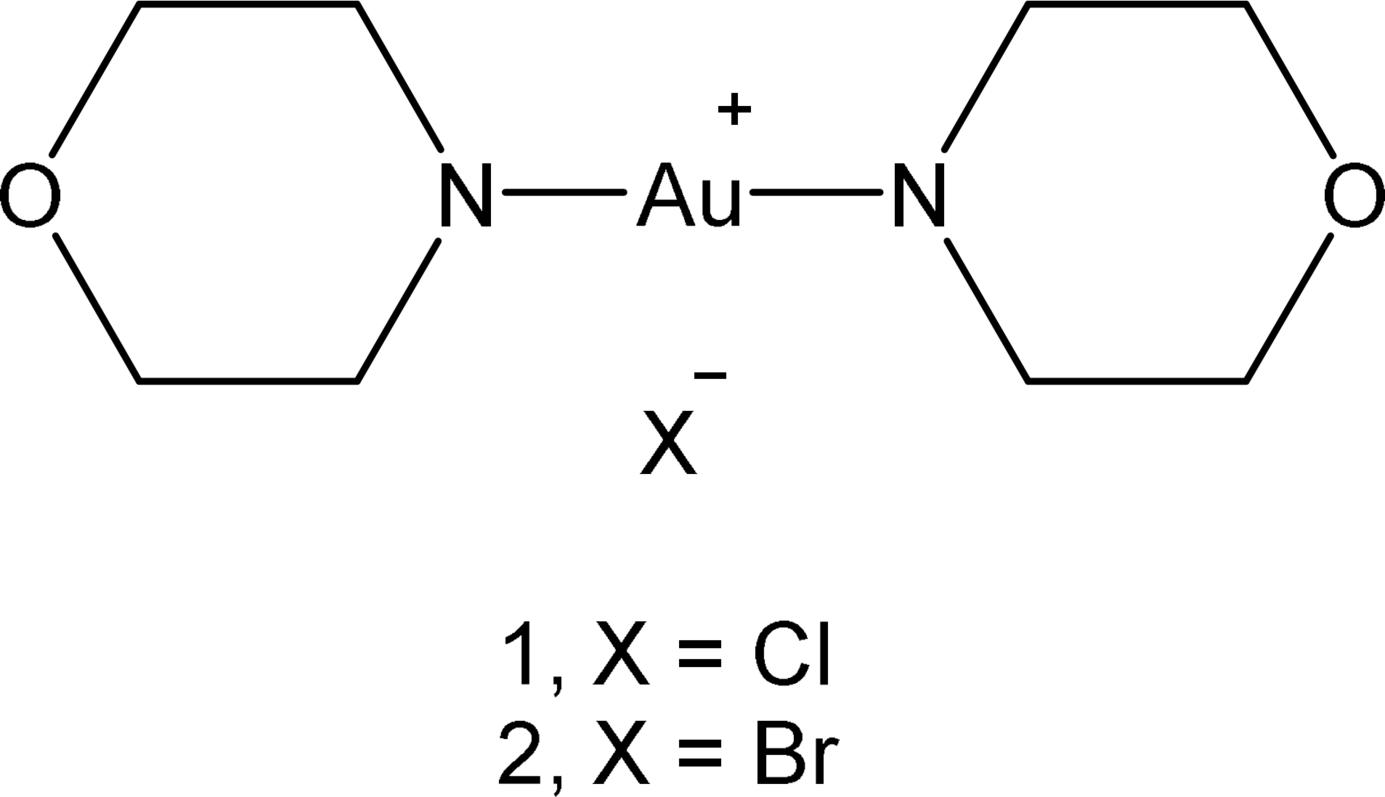


## 2. Structural commentary

At the outset we comment that, for structures that contain more than one residue in the asymmetric unit, the distinction between the categories ‘Structural commentary’ (which generally refers to the asymmetric unit) and ‘Supra­molecular features’ becomes blurred.

**Figure 1:**
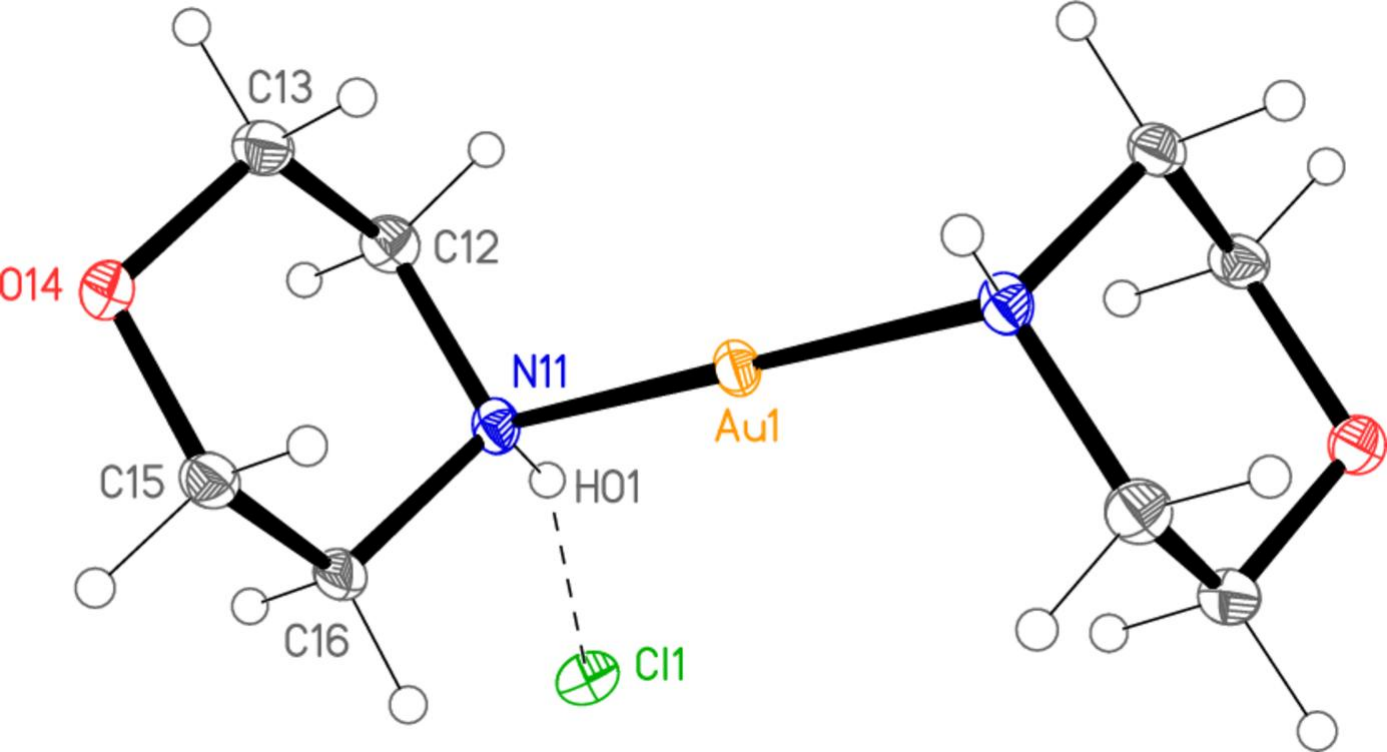
The structure of compound **1** in the crystal, with ellipsoids at the 50% probability level. The asymmetric unit (labelled) is extended over the inversion centre at the gold atom. The dashed line represents the hydrogen bond.

Compounds **1** and **2** crystallize isotypically in space group *C*2/*c* with *Z* = 4. The gold atoms lie on inversion centres at (0.5, 0.5, 0.5) and the halide ions on twofold axes at (0.5, *y*, 0.75). Figs. 1 ▸ and 2 ▸ show the formula units, extended appropriately over the inversion centres. Selected mol­ecular dimensions are presented in Tables 1 ▸ and 2 ▸. The Au—N bond lengths of 2.0631 (19) in **1** and 2.0598 (18) Å in **2** may be considered normal. The coordination geometry at gold is exactly linear by symmetry. Within the asymmetric units, a classical hydrogen bond connects the NH group and the halide ion. The morpholine rings are mutually rotated as viewed along the N11⋯N11^i^ vector, with C12—N11⋯N11^i^—C12^i^ = 180° by symmetry and C16—N11⋯N11^i^—C12^i^ = 56.6 (2)° for **1** and 55.8 (2)° for **2**.

One notable feature is the axial disposition of the gold centres at the morpholine ring, associated with C—C—N—Au torsion angles of around 68°. This conformation is usually regarded as unfavourable for a single substituent of a six-membered ring in the chair form; one would expect the conformation to be equatorial, with an anti­periplanar sequence C—C—N—Au, as was indeed observed for the piperidine complexes in our previous paper (Döring & Jones, 2023 ▸). See also Section 4.

## 3. Supra­molecular features

Hydrogen bonds for **1** and **2** are presented in Tables 3 ▸ and 4 ▸ respectively.

For compound **1**, the chloride ion accepts hydrogen bonds from two symmetry-equivalent NH donors (one in the asymmetric unit and the other with operator 1 − *x*, *y*, 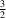 − *z*); the H⋯Cl⋯H angle is 93.9 (12)°. The gold atom is involved in two symmetry-equivalent Au1⋯Cl1 contacts of 3.7187 (5) Å (with operators *x*, 1 + *y*, *z* and 1 − *x*, −*y*, 1 − *z* for the chlorine atoms), with a Cl⋯Au⋯Cl angle of 180° by symmetry; the corresponding Au⋯Cl⋯Au angle is 98.93 (2)° (with operators *x*, −1 + *y*, *z* and *x*, −*y*, 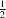 + *z* for the gold atoms).

**Figure 3:**
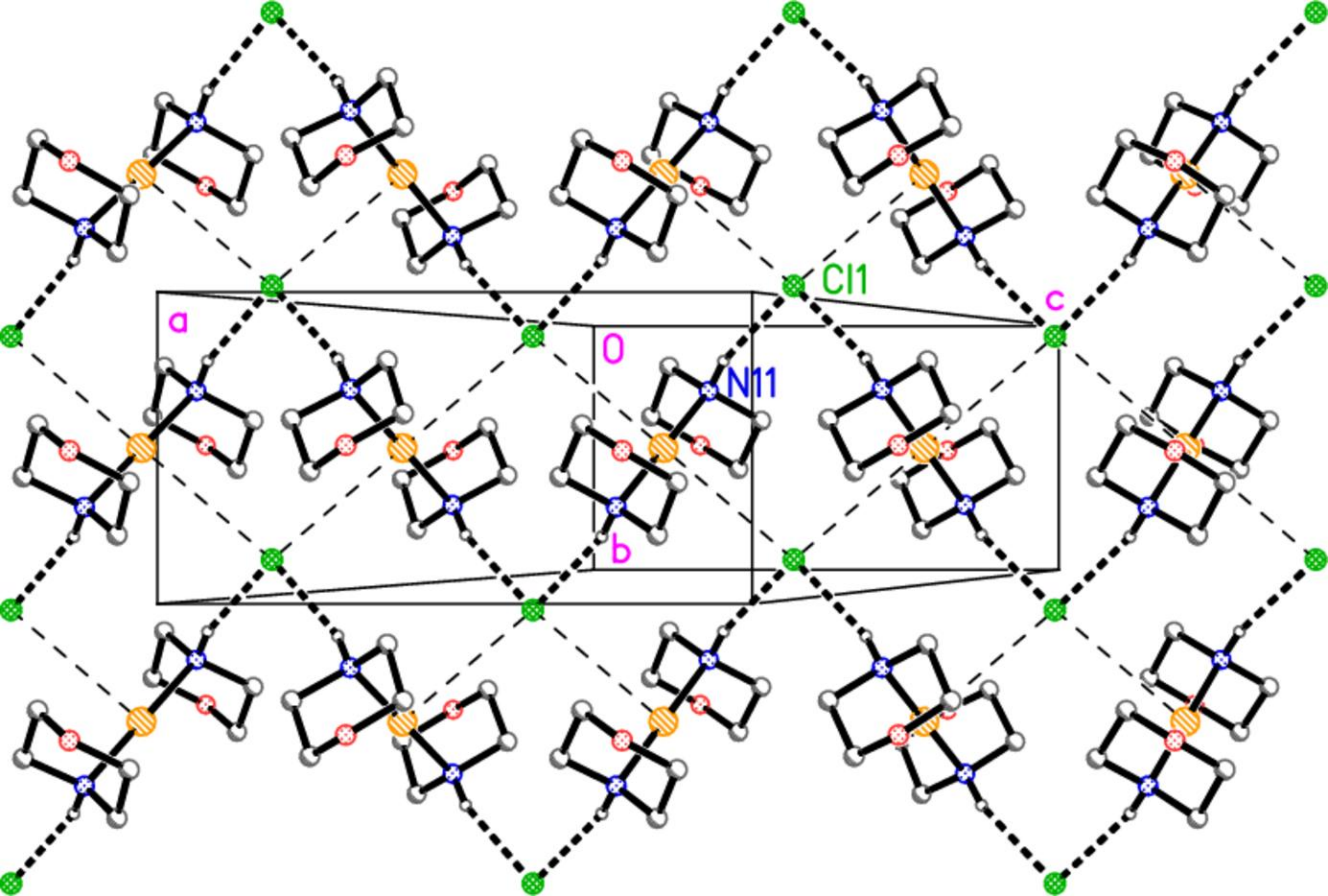
Packing diagram of compound **1**, viewed perpendicular to the *bc* plane in the region *x* ≃ 0.5. Hydrogen atoms bonded to carbon are omitted for clarity. Thick dashed lines indicate hydrogen bonds; thin dashed lines indicate Au⋯Cl contacts.

The contacts combine to form a layer structure parallel to the *bc* plane (Fig. 3 ▸) in the region *x* ≃ 0.5. N—H⋯Cl hydrogen-bonded zigzag chains [⋯Cl⋯(morph)—Au—(morph)⋯]_*n*_, with overall direction parallel to the *c* axis, are crosslinked by the Au⋯Cl contacts. Within the layer, the chloride anion is involved in two C—H⋯Cl contacts that might be regarded as borderline ‘weak’ hydrogen bonds. The morpholine ligands project out of the layer to occupy the spaces at *x* ≃ 0.25 and 0.75. The morpholine oxygen atom is not involved in classical hydrogen bonding, but two C—H⋯O contacts connect the morpholine ligands of the layer at *x* ≃ 0.5 to those of adjacent layers at *x* ≃ 0 and 1. The significant role of the C—H⋯O inter­actions is indirectly implied by the fact that bis­(piperidine)­gold(I) chloride, which lacks the oxygen atoms in the rings, has a quite different packing, involving inversion-symmetric dimers with NH⋯Cl^−^⋯NH linkages (Ahrens *et al.*, 1999 ▸).

The packing of compound **2** is necessarily strictly analogous to that of **1** (and thus no separate packing diagram is presented for **2**), with contact dimensions Au⋯Br = 3.7686 (2) Å, H⋯Br⋯H = 93.3 (11)° and Au⋯Br⋯Au = 98.33 (1)°. Hydrogen bonds for **2** are presented in Table 4 ▸.

## 4. Database survey

The searches employed the routine ConQuest (Bruno *et al.*, 2002 ▸), part of Version 2022.3.0 of the Cambridge Database (Groom *et al.*, 2016 ▸).

Only four other complexes of gold with morpholine are present in the CSD. Two of these involve our own work: [Au(morph)_2_] [N(SO_2_CH_3_)_2_] (refcode DUHKAY; Ahrens *et al.*, 2000 ▸) and (morph)AuCN (FIMSUR; Döring & Jones, 2013 ▸). The third is a cationic complex in the salt [Au(morph)(phosphine)][B(C_6_F_5_)_4_] (OSOZUS; Hesp & Stradiotto, 2010 ▸) whereas the last is the neutral gold(III) complex *trans*-[AuCl_2_(morph)Ph] (WALQOR; Lavy *et al.*, 2010 ▸).

A search for morpholine complexes of any transition metal gave 120 hits that included atom coordinates. A total of 117 structures displayed absolute C—C—N—TM torsion angles of 160–180° (*i.e*. with the metal atom equatorial to the morpholine ring), whereas just six lay in the range 68–78°, representing an axial position for the metal residue (with seven further cases in the range 78–90°, but none with angles < 68°; because of structures containing more than one morpholine and/or differing torsion angles, the sum of these exceeds the number of hits). All six axial systems (DUHKAY, Ahrens *et al.*, 2000 ▸; FIMSUR, Döring & Jones, 2013 ▸; ICADIB, Miller *et al.*, 2011 ▸; REZKUE, Wang & Lian, 2013 ▸; YUXWUK and YUXXAR, Wölper *et al.*, 2010 ▸) involved the coinage metals. We made similar observations for piperidine complexes in the CSD (Döring & Jones, 2023 ▸). It is unclear whether the generally lower coordination numbers of these metals, especially silver and gold, might promote the axial geometry (by reducing steric repulsions), whether electronic effects may play a role, or whether packing effects are involved.

**Figure 4:**
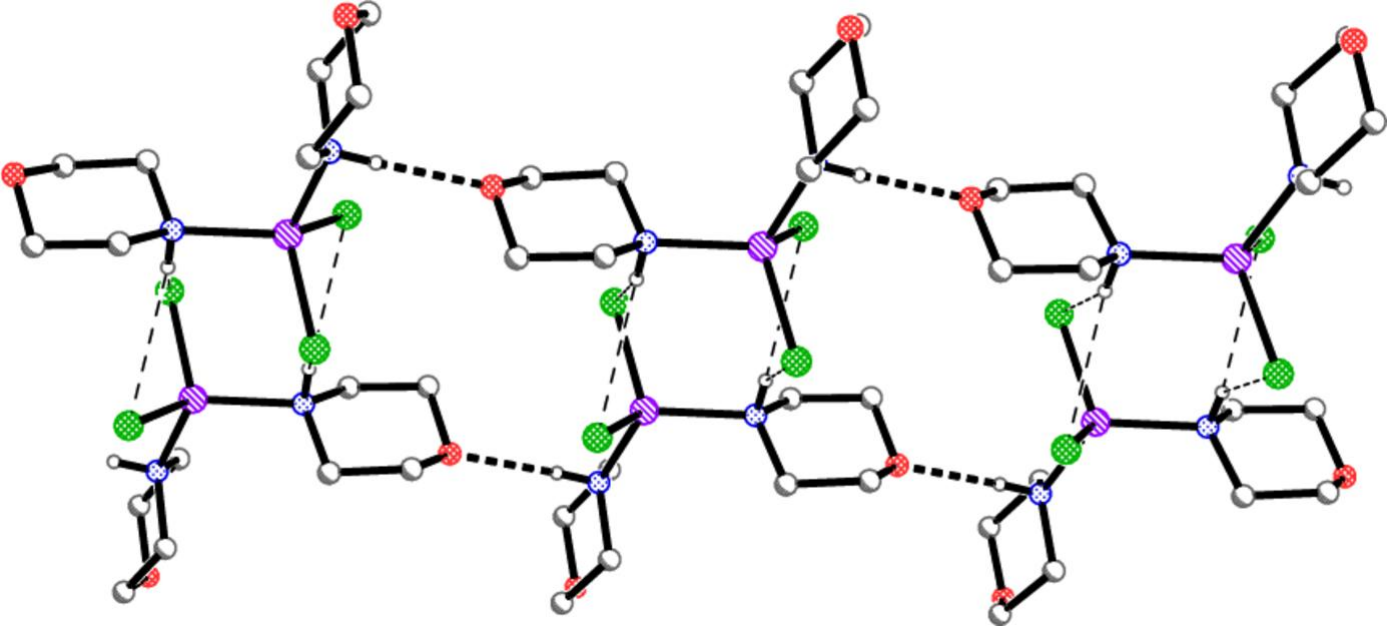
Packing diagram of di­chlorido­bis­(morpholine)­zinc (WIQRIA; Kinens *et al.*, 2018 ▸), drawn using *XP* (Siemens, 1994 ▸) from the deposited coordinates. Dashed lines represent N—H⋯O (thick) or three-centre N—H⋯Cl (thin) hydrogen bonds. Colour codes: Cl green, Zn violet, O red, N blue.

A search for any structure containing morpholine (including those with four-coordinated nitro­gen, but only where the NH function is retained) gave 766 hits. All necessarily contained an NH group, and 378 an additional OH group. Only 144 structures displayed an N—H⋯O_morpholine_ or O—H⋯O_morpholine_ contact shorter then the sum of the van der Waals radii (2.68 Å in the CCDC system), and only 83 of these had a short H⋯O contact < 2.2 Å. This of course merely confirms the general principle that the oxygen atoms of ether groups have a limited tendency to form hydrogen bonds. In an investigation of the frequency of various hydrogen-bonded motifs, Allen *et al.* (1999 ▸) concluded that particular motifs involving oxygen atoms were ‘much less likely to occur if the oxygen atom is two-coordinate’. A typical example, drawn from the hit-list and showing both possible roles of the morpholine oxygen atom, is the complex di­chlorido­bis­(morpholine)­zinc (WIQRIA; Kinens *et al.*, 2018 ▸), with two crystallographically independent morpholine ligands in the mol­ecule, where a short N—H⋯O hydrogen bond (H⋯O 2.08 Å) connects the morpholine NH group of one ligand to the oxygen atom of the other ligand in a neighbouring mol­ecule related by translational symmetry, forming chains of mol­ecules (Fig. 4 ▸). The second independent NH group, however, forms three-centre hydrogen bonds to two chloride ligands of an adjacent chain, whereby the second oxygen atom ‘misses out’ on classical hydrogen-bond formation. We note in passing, after a random check of the hit-list, that the hydrogen bonding is often not discussed in the original references (nor in the corresponding Supplementary Material).

The above searches were limited to structures without disorder. One further relevant structure, which has disordered bridging cyano groups (with alternative orientations C≡N or N≡C), is the polymeric [Ag(CN)(morph)] (CITXAH; Strey & Döring, 2018 ▸). This too has axial positions for the silver atoms at all three independent morpholine ligands, and the packing involves classical N—H⋯N hydrogen bonds and short C—H⋯O contacts, but no N—H⋯O hydrogen bonds.

## 5. Synthesis and crystallization

Single crystals of compound **1** (Ahrens *et al.*, 1999 ▸) were obtained by adding 40 mg (0.125 mmol) of chlorido­(tetra­hydro­thio­phene)­gold(I) to 2 mL of morpholine and overlayering portions of the solution thus obtained with various precipitants. The crystal chosen for structure determination was obtained using petroleum ether. Analysis: calculated C 23.63, H 4.46, N 6.89; found C 23.29, H 4.45, N 6.94%. Crystals of **2** were obtained analogously from 45.6 mg (0.125 mmol) of bromido­(tetra­hydro­thio­phene)­gold(I); again, the measured crystal was obtained using petroleum ether as precipitant.

## 6. Refinement

Crystal data, data collection and structure refinement details are summarized in Table 5 ▸. Structures were refined anisotropically on *F* ^2^. Hydrogen atoms of the NH groups were refined freely [but for **2** with *U*_iso_(H) set to 1.2 × *U*_eq_(N), because the value otherwise refined to below zero]. Methyl­ene hydrogens were included at calculated positions and refined using a riding model with C—H = 0.99 Å and H—C—H = 109.5°, and with *U*_iso_(H) set to 1.2 × *U*_eq_(C).

For compound **2**, an extinction correction was performed using the command ‘EXTI’; the extinction parameter (as defined by *SHELXL*; Sheldrick, 2015 ▸) refined to 0.00023 (3).

## Supplementary Material

Crystal structure: contains datablock(s) 1, 2, global. DOI: 10.1107/S2056989023009702/yz2043sup1.cif

Structure factors: contains datablock(s) 1. DOI: 10.1107/S2056989023009702/yz20431sup2.hkl

Structure factors: contains datablock(s) 2. DOI: 10.1107/S2056989023009702/yz20432sup3.hkl

CCDC references: 2113955, 2113954

Additional supporting information:  crystallographic information; 3D view; checkCIF report

## Figures and Tables

**Figure 2:**
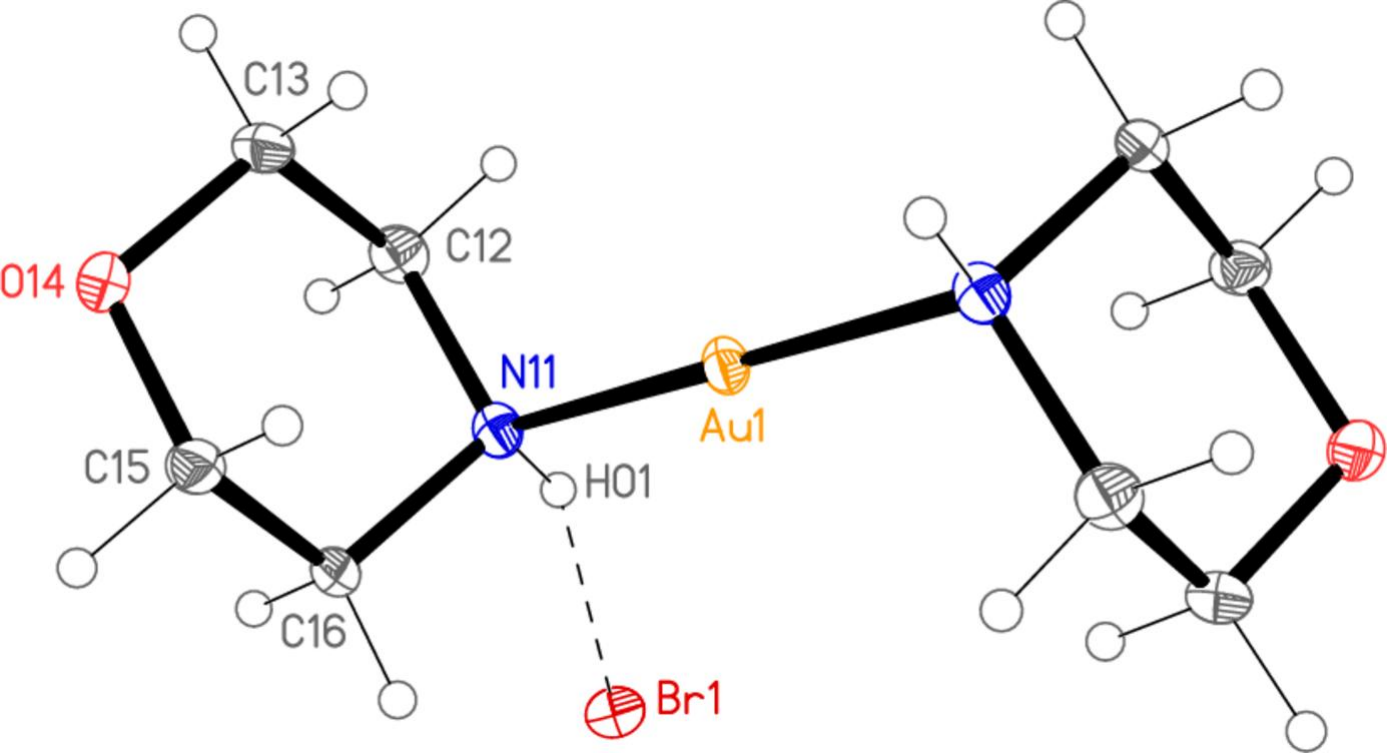
The structure of compound **2** in the crystal, with ellipsoids at the 50% probability level. The asymmetric unit (labelled) is extended over the inversion centre at the gold atom. The dashed line represents the hydrogen bond.

**Table 1:** Selected geometric parameters (Å, °) for **1** [Chem scheme1]

Au1—N11	2.0631 (19)	C13—O14	1.429 (3)
N11—C16	1.491 (3)	O14—C15	1.427 (3)
N11—C12	1.495 (3)		
			
N11—Au1—N11^i^	180.0	C12—N11—Au1	113.41 (13)
C16—N11—C12	108.02 (17)	C15—O14—C13	110.06 (16)
C16—N11—Au1	113.07 (14)		
			
Au1—N11—C12—C13	−68.44 (19)	Au1—N11—C16—C15	67.7 (2)

Symmetry code: (i) 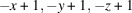 .

**Table 2:** Selected geometric parameters (Å, °) for **2** [Chem scheme1]

Au1—N11	2.0598 (18)	C13—O14	1.427 (2)
N11—C16	1.491 (3)	O14—C15	1.431 (3)
N11—C12	1.491 (3)		
			
N11^i^—Au1—N11	180.00 (7)	C12—N11—Au1	114.42 (13)
C16—N11—C12	107.83 (16)	C13—O14—C15	110.40 (15)
C16—N11—Au1	113.28 (14)		
			
Au1—N11—C12—C13	−68.99 (18)	Au1—N11—C16—C15	68.54 (19)

Symmetry code: (i) 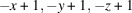 .

**Table 3:** Hydrogen-bond geometry (Å, °) for **1** [Chem scheme1]

*D*—H⋯*A*	*D*—H	H⋯*A*	*D*⋯*A*	*D*—H⋯*A*
N11—H01⋯Cl1	0.86 (3)	2.35 (3)	3.172 (2)	160 (2)
C12—H12*B*⋯Cl1^ii^	0.99	2.92	3.836 (2)	154
C16—H16*A*⋯Cl1^iii^	0.99	2.91	3.654 (2)	132
C13—H13*B*⋯O14^iv^	0.99	2.65	3.511 (3)	146
C15—H15*A*⋯O14^v^	0.99	2.61	3.439 (3)	142

Symmetry codes: (ii) 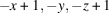 ; (iii) 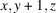 ; (iv) 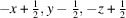 ; (v) 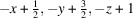 .

**Table 4:** Hydrogen-bond geometry (Å, °) for **2** [Chem scheme1]

*D*—H⋯*A*	*D*—H	H⋯*A*	*D*⋯*A*	*D*—H⋯*A*
N11—H01⋯Br1	0.89 (2)	2.46 (2)	3.3056 (18)	159.0 (19)
C12—H12*B*⋯Br1^ii^	0.99	2.94	3.860 (2)	155
C16—H16*A*⋯Br1^iii^	0.99	2.98	3.717 (2)	132
C13—H13*B*⋯O14^iv^	0.99	2.70	3.542 (3)	144
C15—H15*A*⋯O14^v^	0.99	2.61	3.446 (3)	142

Symmetry codes: (ii) 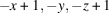 ; (iii) 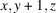 ; (iv) 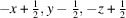 ; (v) 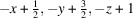 .

**Table 5:** Experimental details

	**1**	**2**
Crystal data		
Chemical formula	[Au(C_4_H_9_NO)_2_]Cl	[Au(C_4_H_9_NO)_2_]Br
*M*_r_	406.66	451.12
Crystal system, space group	Monoclinic, *C*2/*c*	Monoclinic, *C*2/*c*
Temperature (K)	100	100
*a*, *b*, *c* (Å)	18.9504 (9), 5.92161 (19), 11.3049 (5)	18.8719 (6), 6.07840 (17), 11.4050 (4)
β (°)	114.729 (6)	114.595 (4)
*V* (Å^3^)	1152.27 (10)	1189.57 (7)
*Z*	4	4
Radiation type	Mo *K*α	Mo *K*α
μ (mm^−1^)	12.98	15.71
Crystal size (mm)	0.08 × 0.08 × 0.03	0.08 × 0.05 × 0.05

Data collection		
Diffractometer	Oxford Diffraction Xcalibur, Eos	Oxford Diffraction Xcalibur, Eos
Absorption correction	Multi-scan (*CrysAlis PRO*; Rigaku OD, 2022 ▸)	Multi-scan (*CrysAlis PRO*; Rigaku OD, 2022 ▸)
*T*_min_, *T*_max_	0.692, 1.000	0.733, 1.000
No. of measured, independent and observed [*I* > 2σ(*I*)] reflections	18488, 1744, 1440	22827, 1739, 1601
*R*_int_	0.036	0.042
(sin θ/λ)_max_ (Å^−1^)	0.722	0.704

Refinement		
*R*[*F* ^2^ > 2σ(*F* ^2^)], *wR*(*F* ^2^), *S*	0.015, 0.026, 1.06	0.015, 0.026, 1.08
No. of reflections	1744	1739
No. of parameters	70	70
H-atom treatment	H atoms treated by a mixture of independent and constrained refinement	H atoms treated by a mixture of independent and constrained refinement
Δρ_max_, Δρ_min_ (e Å^−3^)	0.72, −0.49	0.58, −0.65

Computer programs: *CrysAlis PRO* (Rigaku OD, 2022 ▸), *SHELXS97* (Sheldrick, 2008 ▸), *SHELXL2018/3* and *SHELXL2019/3* (Sheldrick, 2015 ▸) and *XP* (Siemens, 1994 ▸).

## supplementary crystallographic information

### Bis(morpholine-κN)gold(I) chloride (1) Crystal data

**Table:**

[Au(C_4_H_9_NO)_2_]Cl	*F*(000) = 768
*M**_r_* = 406.66	*D*_x_ = 2.344 Mg m^−^^3^
Monoclinic, *C*2/*c*	Mo *K*α radiation, λ = 0.71073 Å
*a* = 18.9504 (9) Å	Cell parameters from 7694 reflections
*b* = 5.92161 (19) Å	θ = 2.4–30.8°
*c* = 11.3049 (5) Å	µ = 12.98 mm^−^^1^
β = 114.729 (6)°	*T* = 100 K
*V* = 1152.27 (10) Å^3^	Block, colourless
*Z* = 4	0.08 × 0.08 × 0.03 mm

### Bis(morpholine-κN)gold(I) chloride (1) Data collection

**Table:**

Oxford Diffraction Xcalibur, Eos diffractometer	1744 independent reflections
Radiation source: Enhance (Mo) X-ray Source	1440 reflections with *I* > 2σ(*I*)
Detector resolution: 16.1419 pixels mm^-1^	*R*_int_ = 0.036
ω scan	θ_max_ = 30.9°, θ_min_ = 2.4°
Absorption correction: multi-scan (CrysAlisPro; Rigaku OD, 2022)	*h* = −26→27
*T*_min_ = 0.692, *T*_max_ = 1.000	*k* = −8→8
18488 measured reflections	*l* = −16→16

### Bis(morpholine-κN)gold(I) chloride (1) Refinement

**Table:**

Refinement on *F*^2^	Primary atom site location: structure-invariant direct methods
Least-squares matrix: full	Secondary atom site location: difference Fourier map
*R*[*F*^2^ > 2σ(*F*^2^)] = 0.015	Hydrogen site location: mixed
*wR*(*F*^2^) = 0.026	H atoms treated by a mixture of independent and constrained refinement
*S* = 1.06	*w* = 1/[σ^2^(*F*_o_^2^) + (0.0016*P*)^2^ + 3.0402*P*] where *P* = (*F*_o_^2^ + 2*F*_c_^2^)/3
1744 reflections	(Δ/σ)_max_ < 0.001
70 parameters	Δρ_max_ = 0.72 e Å^−^^3^
0 restraints	Δρ_min_ = −0.48 e Å^−^^3^

### Bis(morpholine-κN)gold(I) chloride (1) Special details

**Table:**

Geometry. Non-bonded contacts: 3.7187 (0.0005) Au1 - Cl1_$2 3.7187 (0.0005) Au1 - Cl1_$1 Dihedral angles: 180.00 ( 0.00) C12 - N11 - N11_$5 - C12_$5 56.59 ( 0.22) C16 - N11 - N11_$5 - C12_$5 Copntact angles: 93.91 ( 1.20) H01 - Cl1 - H01_$6 98.93 ( 0.02) Au1_$7 - Cl1 - Au1_$8 180.00 Cl1_$1 - Au1 - Cl1_$2 Symmetry operators: EQIV $1 -x+1, -y, -z+1 EQIV $2 x, y+1, z EQIV $5 1-x,1-y,1-z EQIV $6 1-x, y, 1.5-z EQIV $7 x, -1+y, z EQIV $8 x, -y, 0.5+z

### Bis(morpholine-κN)gold(I) chloride (1) Fractional atomic coordinates and isotropic or equivalent isotropic displacement parameters (Å^2^)

**Table:**

	*x*	*y*	*z*	*U*_iso_*/*U*_eq_	
Au1	0.500000	0.500000	0.500000	0.01184 (3)	
Cl1	0.500000	−0.09186 (14)	0.750000	0.01674 (15)	
N11	0.42478 (11)	0.2880 (3)	0.53588 (18)	0.0123 (4)	
H01	0.4520 (14)	0.179 (4)	0.583 (2)	0.013 (6)*	
C12	0.36225 (13)	0.1930 (4)	0.4149 (2)	0.0154 (4)	
H12A	0.333280	0.074808	0.438030	0.018*	
H12B	0.385631	0.123171	0.360087	0.018*	
C13	0.30752 (14)	0.3799 (4)	0.3402 (2)	0.0159 (5)	
H13A	0.336351	0.492262	0.312681	0.019*	
H13B	0.265864	0.316043	0.260689	0.019*	
O14	0.27328 (8)	0.4903 (3)	0.41568 (14)	0.0164 (3)	
C15	0.33247 (13)	0.5800 (4)	0.5321 (2)	0.0147 (4)	
H15A	0.308067	0.654884	0.583911	0.018*	
H15B	0.362484	0.695237	0.509131	0.018*	
C16	0.38698 (13)	0.3975 (4)	0.6133 (2)	0.0133 (4)	
H16A	0.427037	0.464101	0.693674	0.016*	
H16B	0.357640	0.283561	0.638690	0.016*	

### Bis(morpholine-κN)gold(I) chloride (1) Atomic displacement parameters (Å^2^)

**Table:**

	*U*^11^	*U*^22^	*U*^33^	*U*^12^	*U*^13^	*U*^23^
Au1	0.01025 (5)	0.01374 (5)	0.01282 (5)	0.00112 (5)	0.00610 (4)	0.00242 (5)
Cl1	0.0190 (4)	0.0122 (3)	0.0155 (4)	0.000	0.0037 (3)	0.000
N11	0.0111 (9)	0.0126 (9)	0.0139 (9)	0.0037 (8)	0.0059 (8)	0.0035 (7)
C12	0.0174 (11)	0.0133 (11)	0.0162 (11)	−0.0020 (9)	0.0077 (10)	−0.0026 (9)
C13	0.0166 (11)	0.0185 (12)	0.0133 (11)	−0.0020 (9)	0.0068 (10)	−0.0021 (9)
O14	0.0120 (7)	0.0227 (8)	0.0139 (7)	0.0029 (7)	0.0049 (6)	0.0017 (7)
C15	0.0157 (11)	0.0164 (10)	0.0130 (10)	0.0028 (9)	0.0069 (9)	−0.0003 (9)
C16	0.0135 (11)	0.0163 (11)	0.0112 (10)	0.0019 (9)	0.0061 (9)	0.0022 (9)

### Bis(morpholine-κN)gold(I) chloride (1) Geometric parameters (Å, º)

**Table:**

Au1—N11	2.0631 (19)	C12—H12A	0.9900
Au1—N11^i^	2.0631 (19)	C12—H12B	0.9900
N11—C16	1.491 (3)	C13—H13A	0.9900
N11—C12	1.495 (3)	C13—H13B	0.9900
C12—C13	1.510 (3)	C15—H15A	0.9900
C13—O14	1.429 (3)	C15—H15B	0.9900
O14—C15	1.427 (3)	C16—H16A	0.9900
C15—C16	1.511 (3)	C16—H16B	0.9900
N11—H01	0.86 (3)		
			
N11—Au1—N11^i^	180.0	H12A—C12—H12B	108.2
C16—N11—C12	108.02 (17)	O14—C13—H13A	109.2
C16—N11—Au1	113.07 (14)	C12—C13—H13A	109.2
C12—N11—Au1	113.41 (13)	O14—C13—H13B	109.2
N11—C12—C13	109.38 (18)	C12—C13—H13B	109.2
O14—C13—C12	112.27 (18)	H13A—C13—H13B	107.9
C15—O14—C13	110.06 (16)	O14—C15—H15A	109.3
O14—C15—C16	111.59 (19)	C16—C15—H15A	109.3
N11—C16—C15	109.17 (17)	O14—C15—H15B	109.3
C16—N11—H01	105.9 (16)	C16—C15—H15B	109.3
C12—N11—H01	109.0 (17)	H15A—C15—H15B	108.0
Au1—N11—H01	107.0 (16)	N11—C16—H16A	109.8
N11—C12—H12A	109.8	C15—C16—H16A	109.8
C13—C12—H12A	109.8	N11—C16—H16B	109.8
N11—C12—H12B	109.8	C15—C16—H16B	109.8
C13—C12—H12B	109.8	H16A—C16—H16B	108.3
			
C16—N11—C12—C13	57.7 (2)	C13—O14—C15—C16	−58.5 (2)
Au1—N11—C12—C13	−68.44 (19)	C12—N11—C16—C15	−58.7 (2)
N11—C12—C13—O14	−58.2 (2)	Au1—N11—C16—C15	67.7 (2)
C12—C13—O14—C15	57.7 (2)	O14—C15—C16—N11	60.0 (2)

Symmetry code: (i) −*x*+1, −*y*+1, −*z*+1.

### Bis(morpholine-κN)gold(I) chloride (1) Hydrogen-bond geometry (Å, º)

**Table:**

*D*—H···*A*	*D*—H	H···*A*	*D*···*A*	*D*—H···*A*
N11—H01···Cl1	0.86 (3)	2.35 (3)	3.172 (2)	160 (2)
C12—H12*B*···Cl1^ii^	0.99	2.92	3.836 (2)	154
C16—H16*A*···Cl1^iii^	0.99	2.91	3.654 (2)	132
C13—H13*B*···O14^iv^	0.99	2.65	3.511 (3)	146
C15—H15*A*···O14^v^	0.99	2.61	3.439 (3)	142

Symmetry codes: (ii) −*x*+1, −*y*, −*z*+1; (iii) *x*, *y*+1, *z*; (iv) −*x*+1/2, *y*−1/2, −*z*+1/2; (v) −*x*+1/2, −*y*+3/2, −*z*+1.

### Bis(morpholine-κN)gold(I) bromide (2) Crystal data

**Table:**

[Au(C_4_H_9_NO)_2_]Br	*F*(000) = 840
*M**_r_* = 451.12	*D*_x_ = 2.519 Mg m^−^^3^
Monoclinic, *C*2/*c*	Mo *K*α radiation, λ = 0.71073 Å
*a* = 18.8719 (6) Å	Cell parameters from 8239 reflections
*b* = 6.07840 (17) Å	θ = 3.5–30.5°
*c* = 11.4050 (4) Å	µ = 15.71 mm^−^^1^
β = 114.595 (4)°	*T* = 100 K
*V* = 1189.57 (7) Å^3^	Block, colourless
*Z* = 4	0.08 × 0.05 × 0.05 mm

### Bis(morpholine-κN)gold(I) bromide (2) Data collection

**Table:**

Oxford Diffraction Xcalibur, Eos diffractometer	1739 independent reflections
Radiation source: Enhance (Mo) X-ray Source	1601 reflections with *I* > 2σ(*I*)
Detector resolution: 16.1419 pixels mm^-1^	*R*_int_ = 0.042
ω scan	θ_max_ = 30.0°, θ_min_ = 2.4°
Absorption correction: multi-scan (CrysAlisPro; Rigaku OD, 2022)	*h* = −26→26
*T*_min_ = 0.733, *T*_max_ = 1.000	*k* = −8→8
22827 measured reflections	*l* = −16→16

### Bis(morpholine-κN)gold(I) bromide (2) Refinement

**Table:**

Refinement on *F*^2^	Secondary atom site location: difference Fourier map
Least-squares matrix: full	Hydrogen site location: mixed
*R*[*F*^2^ > 2σ(*F*^2^)] = 0.015	H atoms treated by a mixture of independent and constrained refinement
*wR*(*F*^2^) = 0.026	*w* = 1/[σ^2^(*F*_o_^2^) + (0.0064*P*)^2^ + 1.529*P*] where *P* = (*F*_o_^2^ + 2*F*_c_^2^)/3
*S* = 1.08	(Δ/σ)_max_ = 0.001
1739 reflections	Δρ_max_ = 0.58 e Å^−^^3^
70 parameters	Δρ_min_ = −0.64 e Å^−^^3^
0 restraints	Extinction correction: SHELXL-2019/3 (Sheldrick, 2015), Fc^*^=kFc[1+0.001xFc^2^λ^3^/sin(2θ)]^-1/4^
Primary atom site location: structure-invariant direct methods	Extinction coefficient: 0.00023 (3)

### Bis(morpholine-κN)gold(I) bromide (2) Special details

**Table:**

Geometry. Non-bonded distances: 3.7686 (0.0002) Au1 - Br1_$2 3.7686 (0.0002) Au1 - Br1_$1 Pseudo torsion angles: 180.00 ( 0.00) C12 - N11 - N11_$5 - C12_$5 55.84 ( 0.21) C16 - N11 - N11_$5 - C12_$5 Contact angles: 93.29 ( 1.11) H01 - Br1 - H01_$6 98.33 ( 0.01) Au1_$7 - Br1 - Au1_$8 180.00 ( 0.00) Br1_$1 - Au1 - Br1_$2 Operators for generating equivalent atoms: $1 -x+1, -y, -z+1 $2 x, y+1, z $5 -x+1, -y+1, -z+1 $6 -x+1, y, -z+3/2 $8 x, -y, z+1/2

### Bis(morpholine-κN)gold(I) bromide (2) Fractional atomic coordinates and isotropic or equivalent isotropic displacement parameters (Å^2^)

**Table:**

	*x*	*y*	*z*	*U*_iso_*/*U*_eq_	
Au1	0.500000	0.500000	0.500000	0.01205 (4)	
Br1	0.500000	−0.09457 (5)	0.750000	0.01529 (7)	
N11	0.42269 (10)	0.2957 (3)	0.53179 (17)	0.0124 (4)	
H01	0.4507 (13)	0.183 (4)	0.578 (2)	0.015*	
C12	0.36012 (12)	0.2040 (4)	0.4122 (2)	0.0153 (4)	
H12A	0.330978	0.088952	0.435159	0.018*	
H12B	0.383632	0.135900	0.357941	0.018*	
C13	0.30529 (12)	0.3856 (4)	0.33819 (19)	0.0154 (4)	
H13A	0.334174	0.494886	0.310626	0.018*	
H13B	0.263473	0.323130	0.259703	0.018*	
O14	0.27109 (9)	0.4933 (3)	0.41320 (14)	0.0163 (3)	
C15	0.33036 (12)	0.5800 (4)	0.52921 (19)	0.0148 (4)	
H15A	0.305701	0.652157	0.580629	0.018*	
H15B	0.360713	0.692559	0.507110	0.018*	
C16	0.38456 (11)	0.4011 (4)	0.60869 (19)	0.0130 (4)	
H16A	0.424643	0.464628	0.688740	0.016*	
H16B	0.354807	0.289658	0.632976	0.016*	

### Bis(morpholine-κN)gold(I) bromide (2) Atomic displacement parameters (Å^2^)

**Table:**

	*U*^11^	*U*^22^	*U*^33^	*U*^12^	*U*^13^	*U*^23^
Au1	0.01062 (6)	0.01299 (6)	0.01400 (6)	0.00095 (5)	0.00659 (4)	0.00242 (5)
Br1	0.01822 (15)	0.01134 (14)	0.01438 (14)	0.000	0.00487 (12)	0.000
N11	0.0131 (9)	0.0111 (9)	0.0142 (9)	0.0036 (7)	0.0070 (7)	0.0036 (7)
C12	0.0175 (11)	0.0129 (11)	0.0165 (10)	−0.0027 (9)	0.0079 (9)	−0.0040 (9)
C13	0.0173 (11)	0.0180 (12)	0.0111 (10)	−0.0005 (9)	0.0062 (8)	−0.0017 (9)
O14	0.0121 (7)	0.0216 (8)	0.0146 (7)	0.0032 (7)	0.0049 (6)	0.0006 (7)
C15	0.0154 (10)	0.0162 (10)	0.0125 (10)	0.0026 (9)	0.0057 (8)	−0.0006 (9)
C16	0.0120 (10)	0.0173 (11)	0.0110 (9)	0.0011 (9)	0.0060 (8)	0.0012 (9)

### Bis(morpholine-κN)gold(I) bromide (2) Geometric parameters (Å, º)

**Table:**

Au1—N11^i^	2.0598 (18)	C13—H13A	0.9900
Au1—N11	2.0598 (18)	C13—H13B	0.9900
N11—C16	1.491 (3)	O14—C15	1.431 (3)
N11—C12	1.491 (3)	C15—C16	1.509 (3)
N11—H01	0.89 (2)	C15—H15A	0.9900
C12—C13	1.508 (3)	C15—H15B	0.9900
C12—H12A	0.9900	C16—H16A	0.9900
C12—H12B	0.9900	C16—H16B	0.9900
C13—O14	1.427 (2)		
			
N11^i^—Au1—N11	180.00 (7)	O14—C13—H13B	109.2
C16—N11—C12	107.83 (16)	C12—C13—H13B	109.2
C16—N11—Au1	113.28 (14)	H13A—C13—H13B	107.9
C12—N11—Au1	114.42 (13)	C13—O14—C15	110.40 (15)
C16—N11—H01	107.2 (15)	O14—C15—C16	111.31 (19)
C12—N11—H01	107.6 (16)	O14—C15—H15A	109.4
Au1—N11—H01	106.2 (15)	C16—C15—H15A	109.4
N11—C12—C13	109.65 (18)	O14—C15—H15B	109.4
N11—C12—H12A	109.7	C16—C15—H15B	109.4
C13—C12—H12A	109.7	H15A—C15—H15B	108.0
N11—C12—H12B	109.7	N11—C16—C15	109.18 (16)
C13—C12—H12B	109.7	N11—C16—H16A	109.8
H12A—C12—H12B	108.2	C15—C16—H16A	109.8
O14—C13—C12	112.10 (17)	N11—C16—H16B	109.8
O14—C13—H13A	109.2	C15—C16—H16B	109.8
C12—C13—H13A	109.2	H16A—C16—H16B	108.3
			
C16—N11—C12—C13	58.0 (2)	C13—O14—C15—C16	−58.2 (2)
Au1—N11—C12—C13	−68.99 (18)	C12—N11—C16—C15	−59.1 (2)
N11—C12—C13—O14	−58.0 (2)	Au1—N11—C16—C15	68.54 (19)
C12—C13—O14—C15	57.3 (2)	O14—C15—C16—N11	60.1 (2)

Symmetry code: (i) −*x*+1, −*y*+1, −*z*+1.

### Bis(morpholine-κN)gold(I) bromide (2) Hydrogen-bond geometry (Å, º)

**Table:**

*D*—H···*A*	*D*—H	H···*A*	*D*···*A*	*D*—H···*A*
N11—H01···Br1	0.89 (2)	2.46 (2)	3.3056 (18)	159.0 (19)
C12—H12*B*···Br1^ii^	0.99	2.94	3.860 (2)	155
C16—H16*A*···Br1^iii^	0.99	2.98	3.717 (2)	132
C13—H13*B*···O14^iv^	0.99	2.70	3.542 (3)	144
C15—H15*A*···O14^v^	0.99	2.61	3.446 (3)	142

Symmetry codes: (ii) −*x*+1, −*y*, −*z*+1; (iii) *x*, *y*+1, *z*; (iv) −*x*+1/2, *y*−1/2, −*z*+1/2; (v) −*x*+1/2, −*y*+3/2, −*z*+1.
